# Sodium Nitrate Combined With Dental Pulp Stem Cells Alleviates Atherosclerosis Through Inhibiting Oxidative Stress

**DOI:** 10.1155/sci/5785962

**Published:** 2026-06-12

**Authors:** Zhuang Mao, Han Duan, Zhichao He, Lin Lv, Haichao Yu, Hu Cao, Jingyuan Shao, Xuesong Zhang, Shunying Hu, Hua Wang

**Affiliations:** ^1^ Department of Stem Cell and Regenerative Medicine, Beijing Institute of Radiation Medicine, Beijing, China, irm-cams.ac.cn; ^2^ School of Medicine, Nankai University, Tianjin, China, nankai.edu.cn; ^3^ The Fourth Medical Centre, Chinese People’s Liberation Army General Hospital, Beijing, China, 301hospital.com.cn; ^4^ The Sixth Medical Centre, Chinese People’s Liberation Army General Hospital, Beijing, China, 301hospital.com.cn

**Keywords:** ApoE^−/−^ mice, atherosclerosis, dental pulp stem cell, NaNO_3_, oxidative stress

## Abstract

Atherosclerosis (AS), a leading cause of cardiovascular disease (CVD), is closely associated with excessive oxidative stress. Dietary nitrate has emerged as a promising intervention for cardiovascular protection through the nitrate–nitrite–nitric oxide (NO) pathway. Meanwhile, dental pulp stem cell (DPSC) possess potent antioxidant properties. However, the potential synergistic effect of sodium nitrate (NaNO_3_) and DPSC on AS remains unclear. In this study, ApoE^−/−^ mice were fed a high‐fat diet (HFD) and treated with NaNO_3_ and/or DPSC. The combined treatment markedly attenuated atherosclerotic plaque formation, reduced oxidative stress, increased endothelial NO synthase (eNOS) expression, and decreased circulating monocyte levels. Furthermore, in vitro assays revealed that NaNO_3_ and DPSC synergistically alleviated oxidative stress and promoted macrophage polarization toward the M2 phenotype, thereby suppressing oxidized lipid uptake. Mechanistic studies revealed that these benefits were mediated by activation of the nuclear factor erythroid 2‐related factor 2 (Nrf2) signaling pathway and the subsequent upregulation of heme oxygenase‐1 (HO‐1). Collectively, our findings unveil a novel therapeutic strategy that combines NaNO_3_ with DPSC to alleviate oxidative stress and inflammation, presenting a promising approach for the treatment of AS.

## 1. Introduction

Atherosclerosis (AS), a prevalent cardiovascular disorder primarily affecting middle‐aged and elderly individuals, is recognized as a leading cause of mortality due to its severe pathogenicity and high fatality rate in advanced stages [1]. With ongoing improvements in living standards, the incidence of AS has been rising in recent years, with a notable trend toward earlier onset. An imbalance between oxidative/antioxidant mechanisms leads to oxidative stress and a deficiency in nitric oxide (NO). This imbalance promotes the generation of reactive oxygen species (ROS), which stimulates the expression of cytokines and chemokines, thereby recruiting monocytes to the endothelial layer. Upon migration into the subendothelial space, monocytes differentiate into macrophages. These macrophages engulf oxidatively modified lipoproteins, transforming into foam cells, and continuously release oxidants that exacerbate oxidative stress. Collectively, these processes contribute critically to the progression of AS [2–5]. Although the pathogenesis of AS has been studied for many years and multiple hypotheses exist, the oxidative modification hypothesis—centered on disrupted redox balance—has gained increasing support [6]. Consequently, targeting oxidative stress may represent a promising therapeutic avenue for AS.

NO, a ubiquitous signaling molecule, plays critical roles in vascular homeostasis, immune defense, neurotransmission, and cellular energy metabolism. Through the activation of transcription factors, NO enhances its own bioavailability and reinforces antioxidant capacity in vascular and cardiac tissues. Furthermore, it modulates cellular redox responses to oxidative stress and contributes to antioxidant defense mechanisms. Deficiency or dysregulation in NO signaling is implicated in the pathogenesis of various oxidative stress‐related disorders, including cardiovascular and neurodegenerative diseases [7, 8]. In a recent study, nitrate dietary supplementation was shown to improve endothelial function and atherosclerotic plaque progression in ApoE knockout (ApoE^−/−^) mice fed a high‐fat diet (HFD), mediated via the nitrate–nitrite–NO pathway [9]. Dietary nitrate intake thus promotes cardiovascular health through an alternative NO‐generating route, enhancing NO production independently of classical NO synthase (NOS) activity [10, 11]. Dietary nitrate is converted to nitrite and NO via oral bacterial reductases, gastric acid, and molybdenum enzymes (e.g., xanthine oxidoreductase and aldehyde dehydrogenase) [12].

Mesenchymal stem cells (MSCs), which can be isolated from a variety of tissues such as bone marrow, umbilical cord, placenta, adipose tissue, and dental pulp, possess the capacity for self‐renewal and multilineage differentiation [13]. In pathological conditions, MSCs have been shown to mitigate elevated oxidative stress and reduce ROS, thereby protecting cells from oxidative damage [14, 15]. Dental pulp stem cell (DPSC), a type of MSCs derived from dental pulp, retain similar regenerative properties and functional characteristics as other MSCs. These cells exhibit the advantages of low immunogenicity, ease of isolation, and extensive in vitro expansion [16]. Previous studies have demonstrated that DPSC could counteract oxidative injury through scavenging free radicals and enhancement of cellular antioxidant defenses. Additionally, they have been reported to ameliorate elastase‐induced emphysema and reduce oxidative stress associated with peripheral nerve injury in the brain [17, 18]. These beneficial attributes suggest that DPSC may hold potential for attenuating the progression of AS under HFD conditions [19]. Given the growing interest in antioxidant‐based strategies for AS, DPSC represent a promising candidate for suppressing plaque development via modulation of oxidative stress pathways. Both dietary nitrate and MSCs have emerged as potential antioxidative interventions, though they appear to operate through distinct mechanisms [20, 21]. Although scant research exists on anti‐atherosclerotic therapy via dietary nitrate and DPSC, we scrutinized the impact of dietary NaNO_3_ paired with DPSC on AS, unveiling the cellular and molecular mechanisms underpinning the antioxidant benefits of this combination therapy.

## 2. Materials

### 2.1. Cultivation of DPSC

DPSC was gifted by Beijing SH Biotechnology (http://www.bjshbio.com/). Cells at passages 3–4 were cultured in AM‐V serum‐free medium specifically formulated for MSCs (TBD, Tianjin, China) and incubated at 37°C and 5% CO_2_ for 48 h, and then DPSC and conditioned medium of DPSC (DPSC‐CM) were collected, respectively. DPSCs were used for in vivo animal experiments, while DPSC‐CM was utilized for subsequent in vitro cellular experiments.

### 2.2. RAW264.7 Cell Culture and Experimental Design

The mouse monocyte macrophage RAW264.7 was obtained from the American Type Culture Collection (ATCC), and cultured in Dulbecco’s Modified Eagle Medium (DMEM; Thermo Fisher Scientific, Gaithersburg, MD, USA) supplemented with 10% fetal bovine serum (FBS; Excell Bio, Uruguay) at 37 °C in a humidified atmosphere of 5% CO_2_. RAW264.7 cultured in the abovementioned conditions was defined as M0 macrophages.

To investigate whether oxidative stress could induce ROS production and M1 polarization, RAW264.7 cells were exposed to hydrogen peroxide (H_2_O_2_). RAW264.7 were randomly assigned to five groups: control (Control), H_2_O_2_ alone treatment group (H_2_O_2_), H_2_O_2_ + NaNO_3_ treatment group (NaNO_3_), H_2_O_2_ + DPSC‐CM treatment group (DPSC‐CM), and H_2_O_2_ + NaNO_3_ + DPSC‐CM treatment group (NaNO_3_ + DPSC‐CM). Upon reaching 80% confluence, the cells were treated with 500 μM H_2_O_2_ or 500 μM NaNO_3,_ and/or DPSC‐CM, for 12 h according to group specifications. The mean fluorescence intensity (MFI) and CD86 and ROS were measured by flow cytometry (Becton–Dickinson, Franklin Lakes, NJ, USA).

To further evaluate the combined effect of NaNO_3_ combined with DPSC‐CM on oxidative stress induced by oxidized low‐density lipoprotein (ox‐LDL), RAW264.7 cells were divided into five groups: control group (Control), ox‐LDL treatment group (ox‐LDL), ox‐LDL + NaNO_3_‐treated group (NaNO_3_), ox‐LDL + DPSC‐CM‐treated group (DPSC‐CM), and ox‐LDL + NaNO_3_ + DPSC‐CM‐treated group (NaNO_3_ + DPSC‐CM). Upon reaching 80% confluence, RAW264.7 cells were treated with 50 μg/ml ox‐LDL (bs‐1698 P, Biosynthesis Biotech, Inc., Beijing, China), 500 μM NaNO_3,_ and/or DPSC‐CM for 24 h according to the group design.

### 2.3. Human Aortic Endothelial Cells (HAoEC) Culture and Experimental Design

The HAoECs were purchased from ATCC and cultured in DMEM supplemented with 10% FBS.

To assess intracellular ROS levels, HAoEC were randomly assigned to the following 5 groups: control group (Control), H_2_O_2_‐treated group (H_2_O_2_), H_2_O_2_ + NaNO_3_‐treated group (NaNO_3_), H_2_O_2_ + DPSC‐CM treatment group (DPSC‐CM), and H_2_O_2_ + NaNO_3_ + DPSC‐CM treatment group (NaNO_3_ + DPSC‐CM). When HAoEC reached 80% confluence, they were first treated with 500 μM H_2_O_2_ for 12 h. Subsequently, 500 μM NaNO_3_ and/or DPSC‐CM were added according to the respective group assignments, and treated for another 24 h. ROS levels were detected by flow cytometry. For the analysis of oxidative stress‐related signaling pathways, HAoEC were treated with 500 μM H_2_O_2_, 500 μM NaNO_3,_ and/or DPSC‐CM for 24 h according to the aforementioned groups, after which Western blotting was performed to evaluate the expression of endothelial NOS (eNOS). In a parallel experiment, HAoEC were subjected to the same treatment conditions for 3 h to analyze the expression of nuclear factor erythroid 2‐related factor 2 (Nrf2), heme oxygenase‐1 (HO‐1), glutathione peroxidase 4 (GPX4), and manganese‐containing superoxide dismutase (MnSOD) by Western blotting.

### 2.4. Animals and Treatments

Six‐to‐8‐week‐old male ApoE knockout (ApoE^−/−^) mice were purchased from Beijing Vital River Experimental Animal Technology Co., Ltd. (China). All mice were housed under specific pathogen‐free (SPF) conditions (temperature 22 ± 2°C, humidity 50 ± 10%, and 12  h light/dark cycle) with ad libitum access to food and water. After acclimation, the mice were randomly assigned to five experimental groups (*n* = 10 per group) using a random number table by an investigator not involved in subsequent procedures: (1) control group (NFD): ApoE^−/−^ mice were fed with normal chow and tap water for 12 weeks; (2) high‐fat group (HFD): ApoE^−/−^ mice were fed with high‐fat chow containing 20% fat, 1.254% cholesterol (CHO), and tap water for 12 weeks; and (3) NaNO_3_‐treated group (NaNO_3_): ApoE^−/−^ mice were fed with high‐fat chow containing 20% fat, 1.254% CHO, and 0.1 g/L NaNO_3_ in tap water for 12 weeks; (4) DPSC treatment group (DPSC): ApoE^−/−^ mice were fed with high‐fat chow containing 20% fat and 1.254% CHO and tap water for 12 weeks, and DPSC (1 × 10^6^ cells in 150 µl PBS) was injected by tail vein at weeks 4, 7, and 11; (5) combined treatment group (NaNO_3_ + DPSC): the ApoE^−/−^ mice were fed with a high‐fat chow containing 20% fat, 1.254% CHO, and 0.1 g/L NaNO_3_ in tap water for 12 weeks, and DPSC (1 × 10^6^ cells in 150 µl PBS) was injected by tail vein at Weeks 4, 7, and 11. All surgical interventions, including tail vein injections and blood collection, were performed under isoflurane anesthesia to minimize suffering. At the end of the experiment, mice were euthanized by cervical dislocation under deep anesthesia. Quantitative assessments of aortic plaque burden (by Oil Red O staining) and myocardial infarct size (by TTC staining) were performed by two independent, blinded observers. Any discrepancies in the evaluation were resolved by consensus. All procedures were approved by the Institutional Animal Care and Use Committee of Laboratory Animal Centre (IACUC‐DWZX‐2022−732).

### 2.5. Cell‐Tracking

DPSCs were labeled with the near‐infrared fluorescent lipophilic dye DiR (YEASEN, Shanghai, China, 40757ES25) according to the manufacturer’s instructions, and then DiR‐labeled DPSC were administered via tail vein injection. In vivo distribution of the transplanted cells was monitored using an in vivo imaging system (IVIS) at 24, 48, and 72 h postinjection.

### 2.6. Lipid Analysis

Peripheral blood samples were collected from the medial canthus vein at Weeks 9 and 12 following the initiation of the HFD. Serum was separated by centrifugation and used to measure the concentrations of total CHO, TGs, high‐density lipoprotein (HDL), and LDL according to the manufacturer’s protocols (Changchun Huili Technology Development Co., Ltd, Changchun, China).

### 2.7. Oxidative Stress Index Detection

Peripheral blood samples were collected at Week 12, and serum was separated by centrifugation. The levels of malondialdehyde (MDA) and total SOD activity were determined using the MDA colorimetric assay kit (TBA Method; E‐BC‐K025‐M, Elabscience, Wuhan, China) and the total‐SOD activity assay kit (WST‐1 Method; E‐BC‐K020‐M, Elabscience, Wuhan, China), respectively. The absorbance values for MDA and the total‐SOD were read at 530–540 nm or at 440–460 nm using a microplate reader (Thermo, MA, USA). Final concentrations of MDA and SOD activity were calculated based on the corresponding standard curves.

### 2.8. Flow Cytometry

Peripheral blood and spleen samples were collected at Week 12. Single‐cell suspensions were prepared from both peripheral blood and splenic tissues, followed by erythrocyte lysis using a commercial lysis buffer (BD Pharmingen, San Diego, CA, USA). The cells were then stained with a FITC‐conjugated rat anti‐mouse Ly6C antibody (553104, BD Pharmingen) and an APC‐conjugated rat anti‐mouse CD11b antibody (553312, BD Pharmingen), and immunophenotypes were analyzed by flow cytometry.

RAW264.7 cells were stained with a FITC‐conjugated rat anti‐mouse CD86 antibody (350862; TONBO Biosciences, San Diego, CA, USA) to evaluate M1 macrophage polarization. Intracellular ROS levels were measured using a ROS Detection Kit (S0033S; Beyotime, Shanghai, China), according to the manufacturer’s instructions. The MFI of CD86 and ROS were analyzed by flow cytometry.

Similarly, HAoEC were stained with the Reactive Oxygen Detection Kit, and then MFI of ROS was detected by flow cytometry.

### 2.9. Histological and Immunohistological Evaluation

#### 2.9.1. Gross Oil‐Red Staining

To evaluate atherosclerotic plaque burden, the left ventricle of mice (*n* = 4 per group) was perfused with 10 mL of phosphate‐buffered saline (PBS), and the aortic root, aortic arch, and abdominal aorta were then carefully dissected and harvested. After removal of surrounding adipose and connective tissues, the aortic segments were fixed in 4% neutral buffered formalin (Servicebio Technology, Wuhan, Hubei, China) at room temperature. The location and size of aortic plaques were determined by Oil Red O staining, and the Oil Red O‐positive areas were statistically analyzed using ImageJ software.

#### 2.9.2. Immunohistochemical Staining (IHC)

The left ventricles of three additional mice in each group were treated as described above and then paraffin‐embedded and sectioned (5 μm). Hematoxylin Eosin (HE) staining was used to assess the size of plaques in the lumen. IHC was used to assess local oxidative stress status. The sections were blocked with 5% (w/v) BSA and incubated with eNOS primary antibody (1:1000; GB12086−100; Servicebio Technology, Wuhan, Hubei, China) overnight, and then incubated with corresponding HRP‐conjugated Goat Anti‐Mouse IgG (1:1000; GB23301; Servicebio Technology, Wuhan, Hubei, China).

#### 2.9.3. Immunofluorescent Staining

Tissue sections were incubated overnight at 4°C in a humidified chamber with a primary antibody against iNOS (1:1000; GB11119−100; Servicebio Technology). Following thorough washing, the sections were incubated with a CY3‐conjugated goat anti‐rabbit IgG secondary antibody (1:300; GB21303; Servicebio Technology) for 1  h at room temperature. After final washes and mounting, images were acquired using an inverted fluorescence microscope (Leica DM6000; Wetzlar, Germany).

### 2.10. TTC Staining

Immediately after the animals were sacrificed, the heart tissue was taken and placed in a clean petri dish, wrapped with OCT embedding agent (Servicebio Technology, G6059, Wuhan, Hubei, China), and snap‐frozen at –20°C for 60 min. Subsequently, the embedded tissues were sectioned into 2–3 mm thick slices. TTC staining solution was added to submerge the tissue slices and incubated for about 30 min in a 37°C water bath in the dark. When the deep red color appeared in the tissue, the TTC staining solution was poured out, and the staining reaction was terminated by adding fixative (Servicebio Technology, G1101, Wuhan, Hubei, China) slowly to submerge the tissue slices. The slices were photographed, and the percentage of the infarcted area was analyzed by Image‐Pro Plus 6.0 software.

### 2.11. Western Blotting Analysis

HAoEC were collected and lysed using RIPA (Beyotime Shanghai, China) buffer supplemented with protease and phosphatase inhibitors. The protein concentration was determined using the BCA assay kit. A total of 20 mg protein was subjected to 10% SDS–PAGE electrophoresis, which was then transferred to a PVDF membrane where it was blocked with 5% skimmed milk and reacted with the primary antibody against β‐actin (Rabbit, 1:10000, ZEN‐BIOSCIENCE, China, 380,624), GAPDH (Rabbit, 1:10000, Abcam, ab181602), HO‐1 (Rabbit, 1:10000, Abcam, ab68477), Nrf2 (Rabbit, 1:10000, CST, 33,649 S), eNOS (Rabbit, 1:3000, Proteintech, 27,120–1‐AP), GPX4 (Rabbit, 1:5000, Proteintech, 30,388–1‐AP), and MnSOD (Rabbit, 1:10000, Proteintech, 24,127–1‐AP) at 4°C overnight. After being thoroughly washed, the membrane was incubated with HRP‐goat‐anti‐rabbit IgG (1:5000, ZB‐2301, ZSGB‐BIO, Beijing) at room temperature for 1 h. The blot was probed with electrochemiluminescence plus reagent (Invitrogen, USA), and band density was estimated via ImageJ software.

### 2.12. Reverse Transcription‐Quantitative Polymerase Chain Reaction (RT‐qPCR)

Total RNA was extracted from aortic tissues using TRIzol reagent (Thermo Fisher Scientific, Inc., MD, USA), and then 2 μg of total RNA was reversed into cDNA using a cDNA reverse transcription kit (Fast All‐in‐one RT Kit, ES Science, Shanghai, China) according to the manufacturers’ instructions. RT‐qPCR was then performed with SybrGreen (Life Technologies, NY, USA) on an ABI 7500 Fast Real‐Time PCR System (Applied Biosystems, Thermo Fisher Scientific, CA, USA). The primer sequences used were as follows: mouse *MMP-9* (*mMMP-9*), sense 5′‐acaagaccctcaggccgtaa‐3′ and antisense 5′‐tagcggtacaagtatgcctgg‐3′; m*iNOS*, sense 5′‐gttctcagcccaacaatacaaga‐3′ and antisense 5′‐gtggacggggtcgatgtcac‐3′; m*Hmox1*, sense 5′‐acagccccaccaagttc‐3′ and antisense 5′‐ggcggtcttagcctcttc‐3′; and m*GAPDH*, sense 5′‐ggcaaagtggagattgttgc‐3′ and antisense 5′‐aatttgccgtgagtggagtc‐3′. *GAPDH* was used as an endogenous reference for normalization.

### 2.13. Cell Oil Red O Staining

RAW264.7 cells were treated with Ox‐LDL (50 μg/ml) for 24 h to induce foam cell formation. Cells were then fixed with Oil Red O fixative for 30 min, followed by staining with oil red O working solution (Solarbio Life Science, G1262, Beijing, China) for 20 min at room temperature. After washing and counterstaining, images were captured under a light microscope. Lipid accumulation was quantified by measuring the Oil Red O‐positive areas using ImageJ software.

### 2.14. Statistical Analysis

All data are presented as mean ± standard mean error (SEM) and differ with a 95% confidence interval. Multiple group comparisons were performed using one‐way analysis (one‐way ANOVA) followed by Tukey’s *T*‐test and data were analyzed with GraphPad Prism 8.0. *p*  < 0.05 were considered statistically significant.

## 3. Results

### 3.1. Combined Treatment Alleviates Atherosclerotic Lesions

To evaluate the therapeutic effect of NaNO_3_ combined with DPSC on AS, ApoE^−/−^ mice were fed a HFD and either administered NaNO_3_ in their drinking water or injected with DPSC. Following the intravenous administration of DiR‐labeled DPSCs, their systemic biodistribution was monitored using an IVIS spectrum system. In vivo fluorescence imaging revealed distinct DPSC signals localized to the heart and aortic regions within 24 h postinjection, with persistent cell retention observed for up to 72 h (Figure 1A). After treatment, aortic tissues were harvested for Oil Red‐O staining to evaluate lipid deposition (Figure 1B). Quantitative analysis demonstrated that the HFD markedly increased atherosclerotic plaque formation in ApoE^−/−^ mice, whereas both NaNO_3_ and DPSC monotherapies significantly reduced lesion area compared with the HFD group. Notably, the combination of NaNO_3_ and DPSC resulted in a further reduction in plaque area compared with either treatment alone (Figure 1C). Consistent with these findings, HE staining of aortic cross sections revealed that combined therapy markedly alleviated vascular wall thickening and plaque formation (Figure 1D). These findings suggest that the combined administration of NaNO_3_ and DPSC provides superior protection against atherosclerotic lesion development compared with either monotherapy.

Figure 1NaNO_3_ combined with DPSC therapy alleviated atherosclerotic lesions in ApoE^−/−^ mice. (A) In vivo distribution of DiR‐labeled DPSCs at hours 24, 48, and 72 postinjection. (B) Representative images of Oil Red O‐stained aorta. (C) Quantification of atherosclerotic plaque area expressed as the ratio of Oil Red O‐positive area to total aortic area. Data are presented as mean ± SEM. *n* = 4. (D) Representative images of aortic sections stained with HE. Scale bar = 100 μm.  ^∗^ *p* < 0.05,  ^∗∗∗^ *p* < 0.001.

**Figure:**
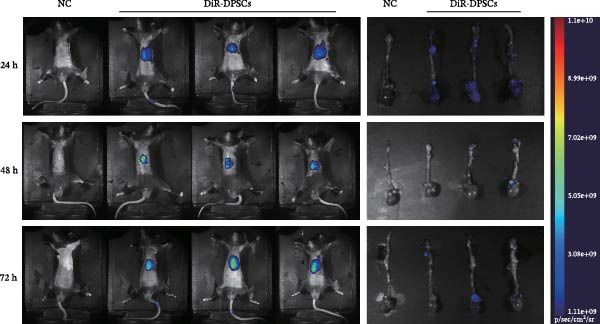
(A)

**Figure:**
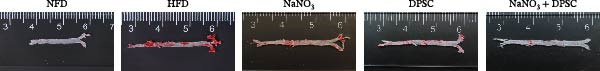
(B)

**Figure:**
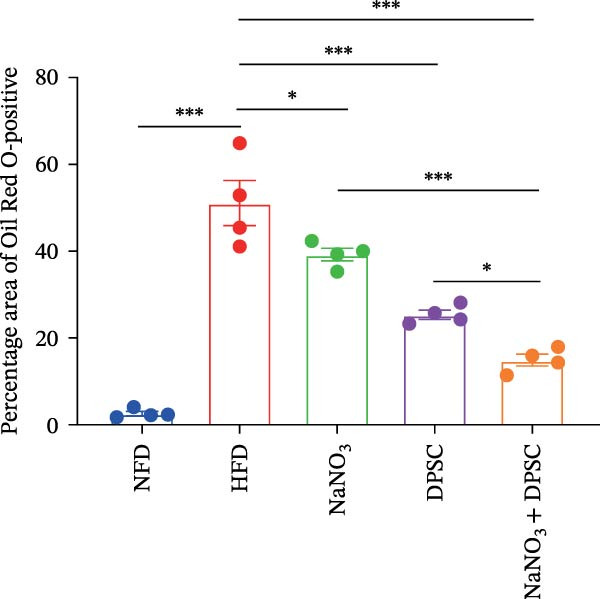
(C)

**Figure:**
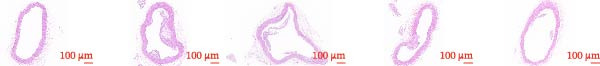
(D)

### 3.2. Combined Treatment Decreases Blood Lipid Level and Alleviates Myocardial Infarction (MI) in HFD‐Fed ApoE^−/−^ Mice

Blood lipid analysis confirmed that HFD feeding successfully induced dyslipidemia, characterized by elevated levels of TGs, total CHO, and LDL. While administration of NaNO_3_ or DPSC alone did not significantly alter these parameters, the combination therapy markedly reduced CHO and LDL levels and significantly increased HDL levels compared with the HFD group (Figure 2A).

Figure 2NaNO_3_ combined with DPSC reduces blood lipid levels and alleviates myocardial infarction in HFD‐fed ApoE^−/−^ mice. (A) Serum concentrations of TG, CHO, LDL, and HDL at weeks 9 and 12. *n* = 4. (B) Representative images of TTC staining of infarcted cardiac tissues. (C) Quantitative analysis of myocardial infarct size. *n* = 3. Data are presented as mean ± SEM.  ^∗^ *p* < 0.05,  ^∗∗^ *p* < 0.01,  ^∗∗∗^ *p* < 0.001.

**Figure:**
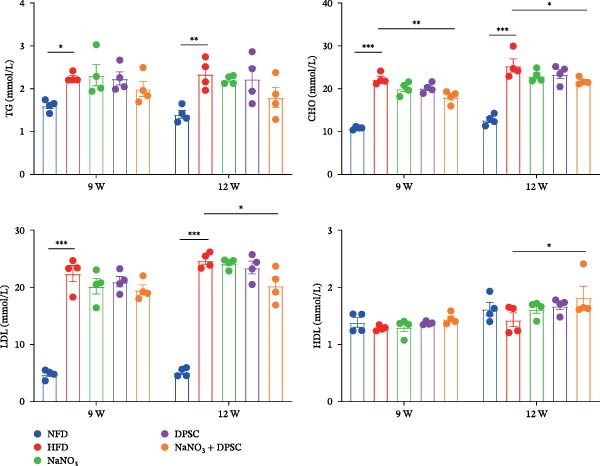
(A)

**Figure:**
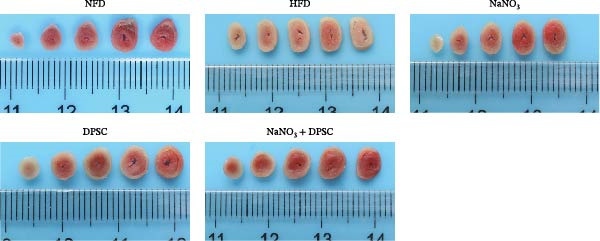
(B)

**Figure:**
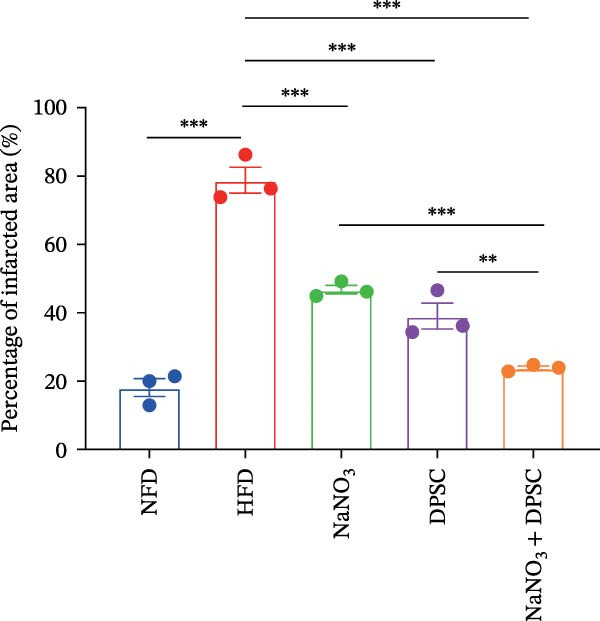
(C)

Given the high susceptibility of ApoE^−/−^ mice to AS‐associated MI, we next evaluated the effects of each treatment on mouse cardiac infarct sizes. HFD feeding induced evident MI, which was partially ameliorated by NaNO_3_ or DPSC monotherapy. Remarkably, the combined treatment further minimized the infarcted area, showing a significant reduction compared with either single treatment (Figure 2B,C). Collectively, these results demonstrate that the combined administration of NaNO_3_ and DPSC not only improves serum lipid profiles but also provides enhanced cardioprotective effects against HFD‐induced myocardial injury in ApoE^−/−^ mice.

### 3.3. Combined Treatment Inhibits Oxidative Stress in HFD‐Fed ApoE^−/−^ Mice

To explore the relationship between AS and oxidative stress, immunofluorescence and immunohistochemistry were performed to examine the oxidative stress marker inducible NOS (iNOS), and the antioxidant enzyme eNOS in aortic tissues. iNOS expression was markedly increased in the HFD group compared with the NFD group, whereas NaNO_3_ or DPSC treatment significantly reduced iNOS levels. Notably, the combined treatment led to a more pronounced decrease in iNOS positivity than either monotherapy (Figure 3A,B). In contrast, eNOS expression was elevated in the NaNO_3_ and DPSC groups compared with HFD‐fed mice, and this enhancement was most evident in the combined treatment group (Figure 3C,D). These findings suggest that the combined administration of NaNO_3_ and DPSC suppresses oxidative stress by downregulating iNOS and upregulating eNOS, thereby contributing to the inhibition of atherosclerotic plaque formation.

Figure 3NaNO_3_ combined with DPSC treatment reduces oxidative stress in the arterial tissue and serum of ApoE^−/−^ mice. (A) Representative immunofluorescence images of iNOS expression in aortic tissues. Scale bar = 50 μm. (B) Quantification of iNOS‐positive cells in aortic tissue. (C) Representative immunohistochemical images of eNOS expression in aortic tissues. Magnification, ×30; scale bar = 50 μm. (D) Quantification of eNOS‐positive cells in aortic tissue. (E) The concentration of MDA and the activity of SOD in serum. (F) The expression levels of *MMP-9*, *iNOS*, and *HO-1* in aortic tissues were detected by RT‐qPCR. Data are presented as mean ± SEM. *n* = 3. 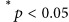, 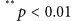, 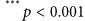.

**Figure:**
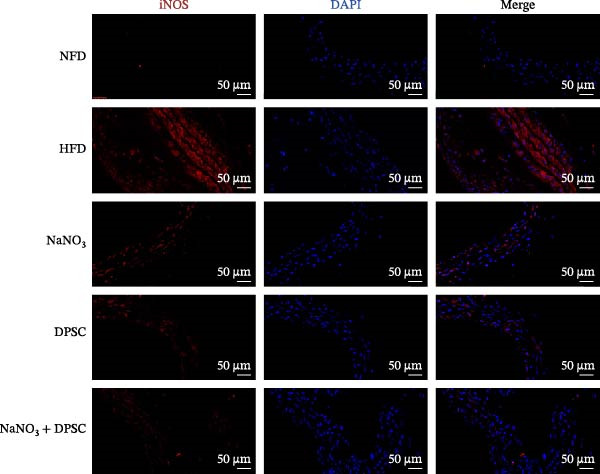
(A)

**Figure:**
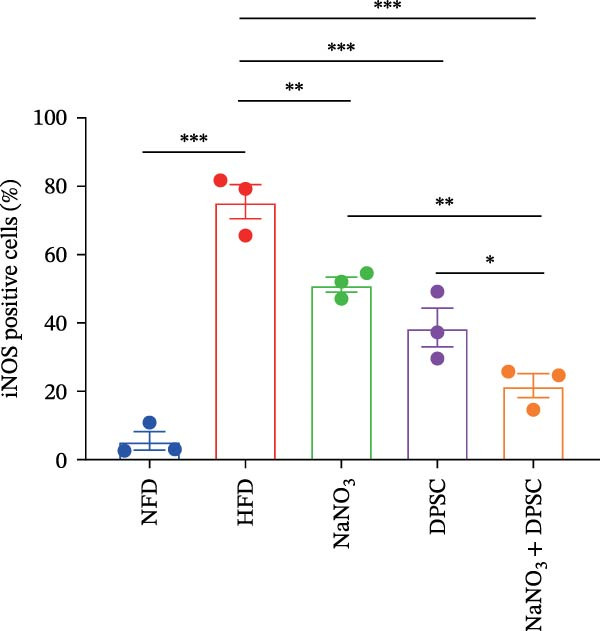
(B)

**Figure:**
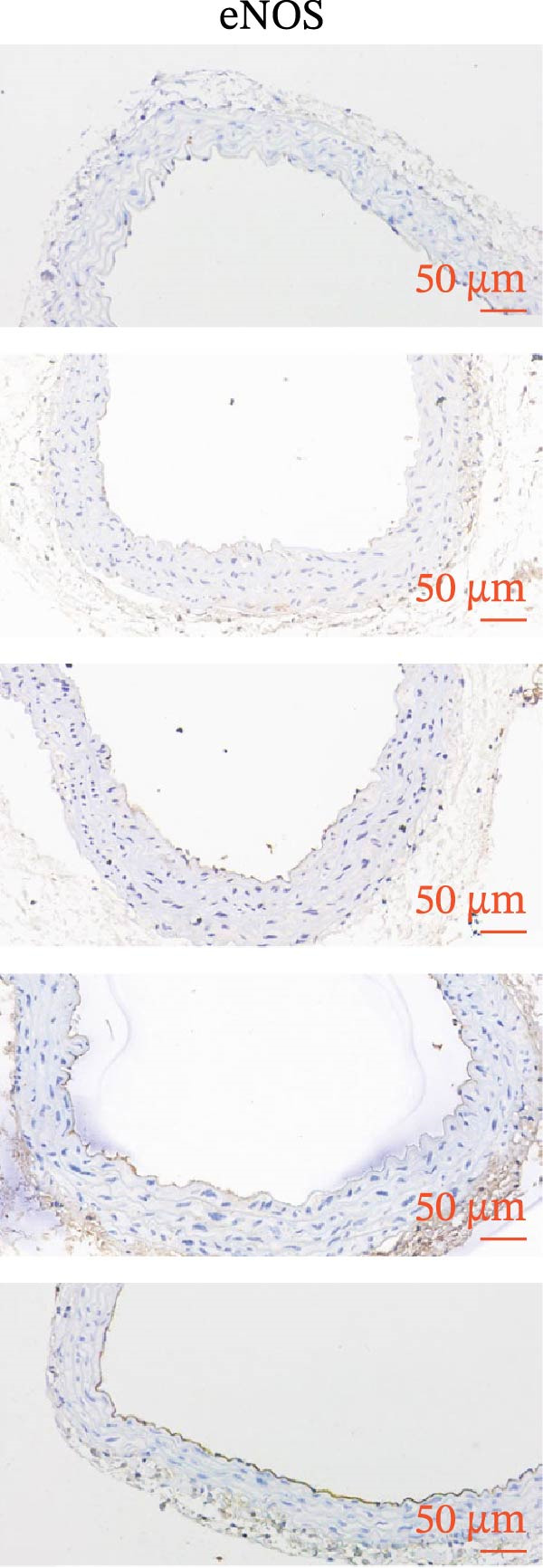
(C)

**Figure:**
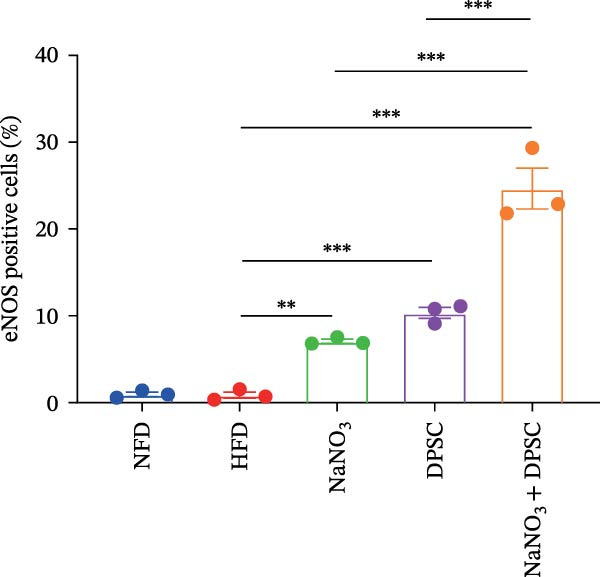
(D)

**Figure:**
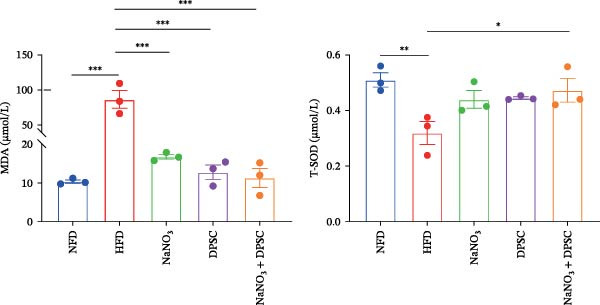
(E)

**Figure:**
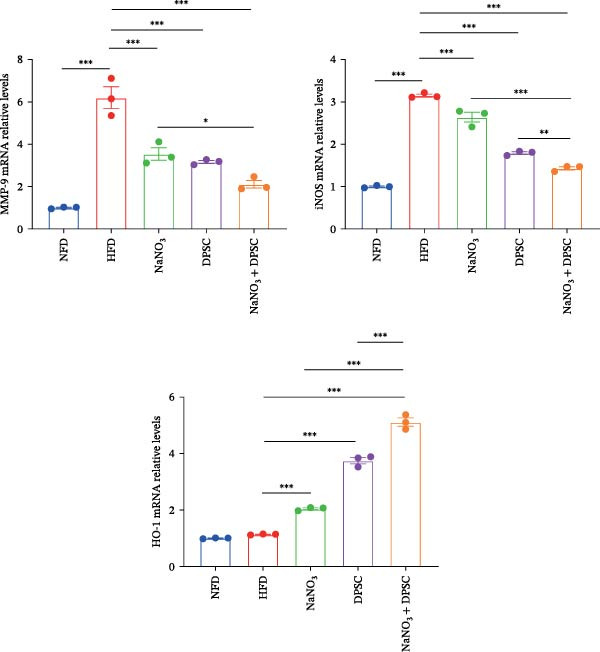
(F)

Blood sampling was conducted post‐experimentation to assess MDA concentration and SOD activity. Findings demonstrated that HFD increased MDA level while reducing SOD potency. Interestingly, treatment with NaNO_3_, DPSC, or their combination reversed these changes, with the combination group showing the greatest reduction in MDA and increase in SOD activity, although the differences did not reach statistical significance (Figure 3E). In addition, RT‐qPCR analysis was performed to assess the expression of oxidative stress–related genes of aortic tissues. Compared with the NFD group, the HFD group exhibited upregulation of the AS‐associated gene *MMP-9* and the oxidative marker *iNOS*, while the antioxidant factor *HO-1* remained unchanged. NaNO_3_ or DPSC monotherapy downregulated *MMP-9* and *iNOS* expression and modestly increased *HO-1* levels, whereas the combined treatment produced the most pronounced effects (Figure 3F). Collectively, these results demonstrate that NaNO_3_ combined with DPSC therapy effectively mitigates HFD‐induced oxidative stress both in aortic tissue and systemically, likely contributing to its protective role against AS progression.

### 3.4. Combined Treatment Downregulates the Percentage of Monocytes in Peripheral Blood and Spleen

Given the crucial role of monocytes in the development of AS, flow cytometry was performed to quantify CD11b^+^ Ly6C^+^ monocytes in peripheral blood and spleen at Week 12. The percentage of these monocytes was significantly elevated in the HFD group compared with the NFD group (67.57 ± 2.15% vs. 19.1 ± 5.5%). Treatment with NaNO_3_ or DPSC alone moderately reduced their proportion (60.47 ± 6.14% and 56.67 ± 5.11%, respectively), although the decrease was not statistically significant. Notably, the combined treatment resulted in a significant reduction in circulating monocytes compared with the HFD group (48.07 ± 4.26% vs. 67.57 ± 2.15%) (Figure 4A,B). A similar trend was observed in the spleen, where monocyte percentages closely mirrored those in peripheral blood (Figure 4C,D).

Figure 4NaNO_3_ combined with DPSC treatment reduces the percentage of monocytes in the peripheral blood and spleen. Peripheral blood cells and splenocytes were stained with anti‐mCD11b and anti‐mLy6C at Week 12 to determine the percentage of CD11b^+^Ly6C^+^ cells. (A) Representative flow cytometry plots of monocytes in peripheral blood. (B) Quantification of the percentage of CD11b^+^Ly6C^+^ monocytes in peripheral blood. (C) Representative flow cytometry plots of monocytes in the spleen. (D) Quantification of the percentage of CD11b^+^Ly6C^+^ monocytes in the spleen. Data are represented as mean± SEM. *n* = 3.  ^∗^ *p* < 0.05,  ^∗∗∗^ *p* < 0.001.

**Figure:**
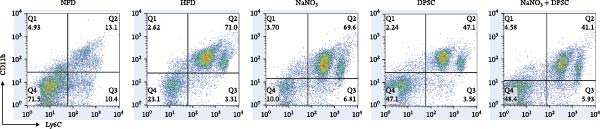
(A)

**Figure:**
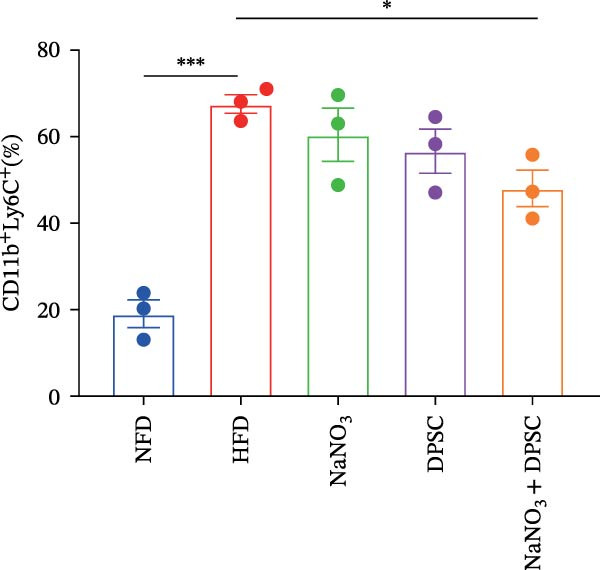
(B)

**Figure:**
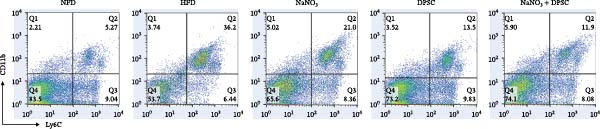
(C)

**Figure:**
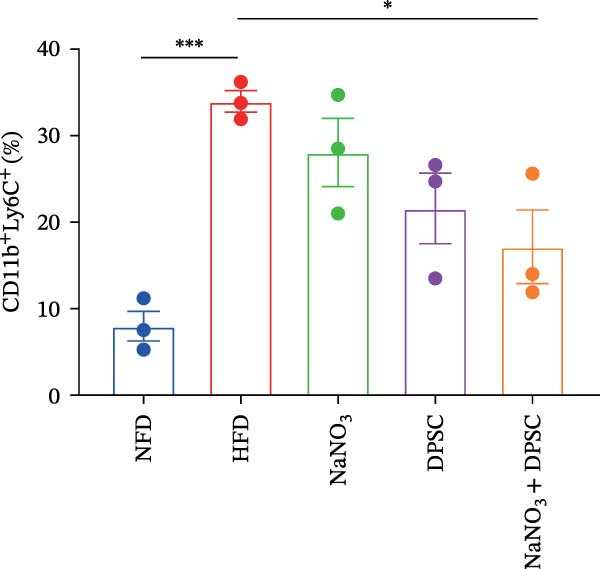
(D)

### 3.5. NaNO_3_ Combined With DPSC‐CM Mitigates the Oxidative Stress of RAW264.7

To evaluate the effects of NaNO_3_ and DPSCs‐CM on macrophage oxidative stress, an in vitro H_2_O_2_‐induced model was established using RAW264.7 cells. Flow cytometry confirmed that H_2_O_2_ treatment markedly increased ROS production and promoted macrophage polarization towards the pro‐inflammatory M1 phenotype, as indicated by elevated CD86 MFI. Treatment with NaNO_3_, DPSC‐CM, or their combination significantly attenuated ROS levels and CD86 expression, with the combined treatment exhibiting the most pronounced inhibitory effects compared with either monotherapy (Figure 5A,B).

Figure 5Effects of NaNO_3_ combined with DPSC‐CM on oxidative stress, M1 polarization, and lipid uptake in H_2_O_2_‐stimulated RAW264.7 cells. RAW264.7 cells were treated with NaNO_3_ and/or DPSCs‐CM for 12 h under 500 μM H_2_O_2_ stimulation. Flow cytometry was performed to detect ROS and the MFI of CD86. (A) Representative images (A1) and quantification (A2) of ROS MFI. (B) Representative image (B1) and statistical graph (B2) of CD86 MFI. (C) Representative Oil‐Red O staining images of RAW264.7 cells. Scale bar = 200 μm (upper, ×10) or 50 μm (lower, ×40). Data are presented as mean ± SEM. *n* = 3.  ^∗^ *p* < 0.05,  ^∗∗^ *p* < 0.01,  ^∗∗∗^ *p* < 0.001.

**Figure:**
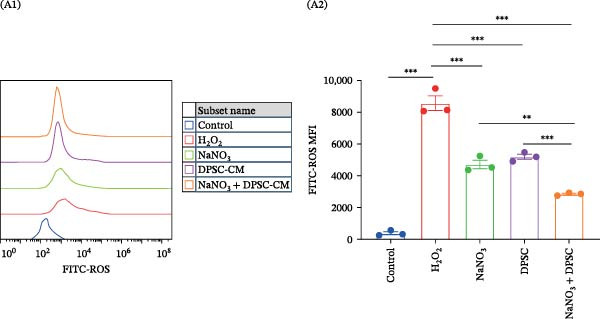
(A)

**Figure:**
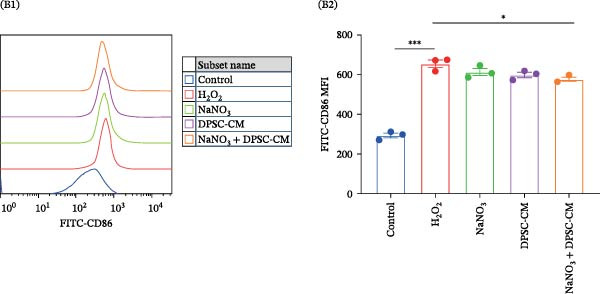
(B)

**Figure:**
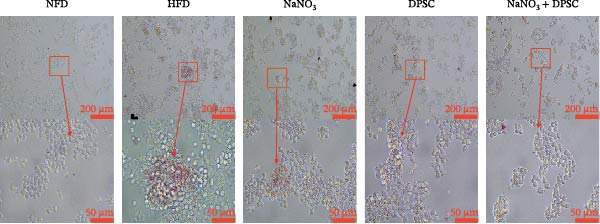
(C)

Inflammatory macrophages can accumulate lipids by continuous phagocytosis, eventually forming foam cells. To assess the impact of the combined treatments on lipid uptake, RAW264.7 cells were exposed to ox‐LDL, and intracellular lipid accumulation was detected by Oil Red O staining. Cells treated with ox‐LDL alone showed substantial lipid droplet accumulation (red staining), whereas treatment with NaNO_3_, DPSC‐CM, or a combination markedly reduced lipid accumulation. Notably, the combined treatment was more effective than either monotherapy (Figure 5C). These results indicate that NaNO_3_ combined with DPSC‐CM can effectively suppress oxidative stress, inhibit M1 polarization, and reduce lipid accumulation in macrophages under oxidative stress conditions, suggesting a synergistic protective effect.

### 3.6. NaNO_3_ Combined With DPSC‐CM Alleviates the Oxidative Stress of HAoEC

Oxidative stress‐induced endothelial dysfunction plays a central role in the progression of AS. To assess the ability of NaNO_3_ and DPSC‐CM to attenuate endothelial oxidative injury, an in vitro oxidative stress model was established by exposing HAoEC to H_2_O_2_. Flow cytometric analysis revealed that ROS production was markedly increased in H_2_O_2_‐treated cells but significantly reduced following treatment with NaNO_3_, DPSC‐CM, or their combination. The combined treatment group exhibited the most pronounced reduction in ROS levels (Figure 6A).

Figure 6NaNO_3_ combined with DPSC‐CM reduces oxidative stress in HAoEC. (A) Representative flow cytometry plots and quantification of ROS. (B) Western blot was used to detect the expression levels of eNOS and statistical graphs. Western blot analysis the expression of Nrf2 (C) and HO‐1 (D) and the quantitative results. Western blot analysis the expression of GPX4 (E) and MnSOD (F), and their quantitative graphs. All data are presented as mean ± SEM. *n* = 3.  ^∗^ *p* < 0.05,  ^∗∗^ *p* < 0.01,  ^∗∗∗^ *p* < 0.001.

**Figure:**
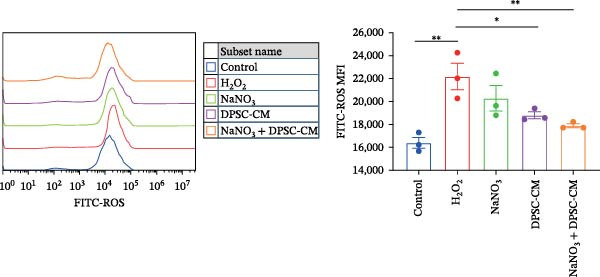
(A)

**Figure:**
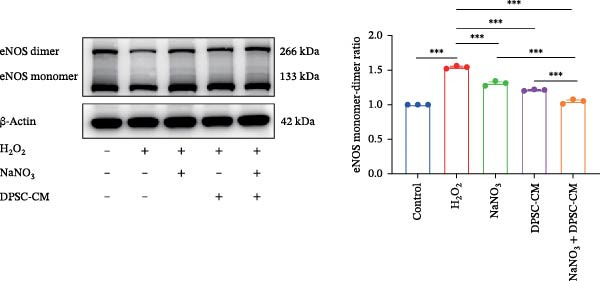
(B)

**Figure:**
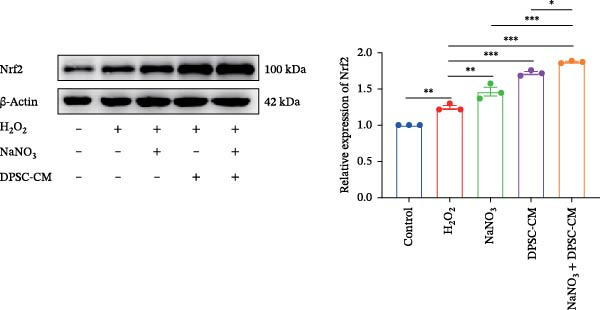
(C)

**Figure:**
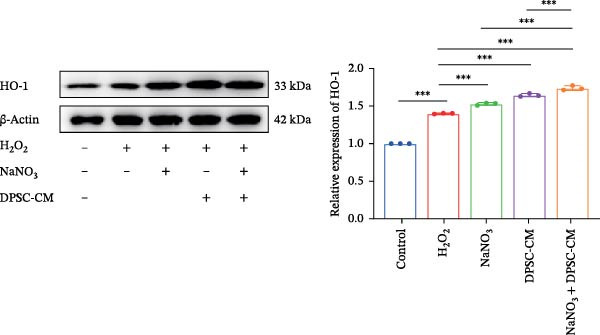
(D)

**Figure:**
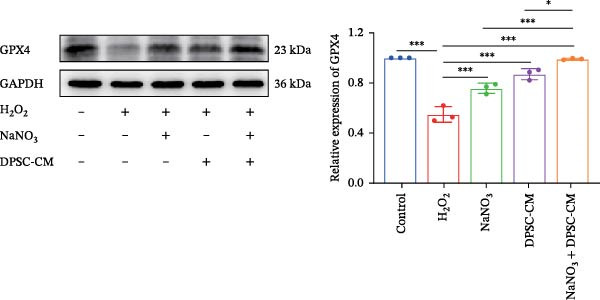
(E)

**Figure:**
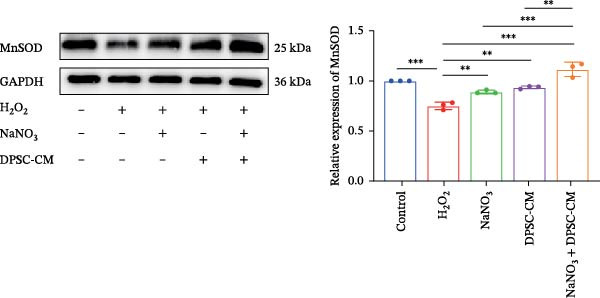
(F)

Western blot analysis demonstrated that the expression of the antioxidant enzyme eNOS was elevated in all treatment groups compared with H_2_O_2_ alone, with the combined group showing the greatest enhancement (Figure 6B).

To further investigate the antioxidant mechanisms, we examined the expression of Nrf2/HO‐1, GPX4, and MnSOD in HAoEC. H_2_O_2_ exposure slightly activated Nrf2/HO‐1 signaling, whereas NaNO_3_, DPSC‐CM, and especially their combination markedly upregulated this pathway (Figure 6C,D). Similarly, the expression of GPX4 and MnSOD was notably increased in all treatment groups, with the most substantial elevation observed under combined treatment (Figure 6E,F). Collectively, these results indicate that NaNO_3_ combined with DPSC‐CM effectively activates the Nrf2/HO‐1 antioxidant pathway and enhances the expression of key antioxidant enzymes (eNOS, GPX4, and MnSOD), thereby mitigating oxidative stress‐induced injury in endothelial cells.

## 4. Discussion

AS‐related cardiovascular mortality escalates due to improved living standards and risks such as obesity and physical inactivity. Substantial evidence supports a central role of oxidative stress in the pathogenesis of AS. Oxidative stress is characterized by a persistent overproduction of ROS that overwhelms endogenous antioxidant defenses [22]. Elevated ROS levels activate the Nrf2/HO‐1 signaling pathway, leading to the upregulation of key antioxidant enzymes, including catalase (CAT), SOD, and glutathione/glutathione synthetase (GSH/GSS) [23]. When ROS generation exceeds the scavenging capacity of these enzymatic systems, redox homeostasis is disrupted, contributing to LDL oxidation, endothelial dysfunction, and foam cell formation—key processes in atherogenesis [24]. Antioxidant stress treatment could be the most efficient strategy for AS.

Recent studies indicate that dietary nitrate supplementation significantly improves endothelial function and attenuates atherosclerotic plaque progression in ApoE^−/−^ mice on an HFD [9, 25], and this is linked to increase NO production [26]. iNOS and eNOS coproduction often accompanies NO production. In this context, a previous chronic AS study demonstrated that genetic deletion of iNOS in ApoE^−/−^ mice led to a significant reduction in plaque formation under HFD feeding, suggesting a pro‐atherogenic role of iNOS‐derived NO [27].

DPSCs exert antioxidant effects that are closely linked to the multifunctional cytoprotective enzyme HO‐1. HO‐1’s effectiveness is due to its degradation of pro‐oxidative heme and neutralization of its byproducts, biliverdin and bilirubin. The activation of Nrf2 and HO‐1 under oxidative stress appears crucial for MSC to resist inflammation and ROS [15, 28]. Consistent with this, HO‐1 induction by MSCs has been shown to mediate antioxidant and anti‐inflammatory protection in multiple disease models, such as radiation‐induced aortic injury, septic lung injury, pancreatitis, and renal injury [29–31].

Our findings suggest that the combination of NaNO_3_ and DPSC represents a promising therapeutic strategy to attenuate the development of atherosclerotic lesions. In ApoE^−/−^ mice fed a HFD, we observed a significant increase in blood lipid levels, consistent with diet‐induced dyslipidemia. While neither DPSC‐CM nor NaNO_3_ alone substantially altered lipid profiles, their combined administration partially reduced lipid deposition. This effect was accompanied by a marked prevention of atherosclerotic plaque formation and mitigation of lesion severity. Previous studies have reported that intravenous administration of MSC‐CM does not influence lipid metabolism [32], and our data align with these observations, further supporting the notion that the anti‐atherosclerotic benefits of the combination therapy are likely mediated through lipid‐independent mechanisms. Notably, the combined treatment reduced monocyte levels in ApoE^−/−^ mice. Given the established role of monocytes as primary precursors of inflammatory macrophages in AS, their reduction may contribute to suppressing key processes such as macrophage infiltration, activation, and foam cell formation, thereby attenuating disease progression.

AS development is related to the monocyte increase, oxidative stress, NO signaling impairment, and bioavailability reduction linked to endothelial dysfunction [33]. NOS, which exists in three isoforms—inducible iNOS, nNOS, and eNOS—plays a central role in NO generation and the maintenance of cardiovascular and metabolic homeostasis. eNOS is active only as a homodimer and produces beneficial NO under normal conditions, whereas iNOS‐derived NO is detrimental under pathologies [34, 35]. Studies indicate that iNOS expression is markedly upregulated under oxidative stress and is associated with multiple stages of cardiovascular disease (CVD). Elevated iNOS activity leads to excessive NO production, which reacts with superoxide to form peroxynitrite (ONOO^−^), a potent oxidant that promotes cellular toxicity, protein nitration, and endothelial injury, thereby accelerating atherosclerotic progression [36]. In the cardiovascular system, eNOS plays a central role in regulating vascular tone and maintaining endothelial homeostasis. Endothelial dysfunction, a well‐established precursor to CVD, can be triggered by reduced eNOS activity or expression [37]. Studies have shown that endothelial dysfunction can be ameliorated by dietary nitrate supplementation, and in addition, the nitrate–nitrite–NO pathway may prevent eNOS uncoupling, restoring eNOS function and alleviating cardiorenal and CVD [38–40]. In the present study, we observed a marked increase in iNOS expression and a concomitant decrease in eNOS levels in aortic tissues of mice fed a HFD, as confirmed by immunofluorescence and immunohistochemical analyses. Notably, the combination of NaNO_3_ and DPSC was found to significantly suppress iNOS upregulation and promote eNOS expression. Further assessment of systemic oxidative stress markers revealed that the combined treatment effectively reduced MDA levels and enhanced the total‐SOD activity, indicating a restoration of redox balance. These results suggest that the potent anti‐atherosclerotic effect of NaNO_3_ and DPSC combination therapy may be mediated, at least in part, through the attenuation of oxidative stress and restoration of NO homeostasis.

Macrophages significantly influence AS progression. Research has indicated that DPSC‐CM can suppress M1 macrophage polarization and promote an M2 anti‐inflammatory phenotype [41]. This study evaluated the antioxidative stress effects of NaNO_3_ and DPSC on macrophages exposed to H_2_O_2_ in an in vitro model. AS formation occurs through two steps; during the first, monocytes migrate to the subendothelium, where they transform into M1 pro‐inflammatory macrophages under oxidative stress. The second stage involves macrophage ingestion of ox‐LDL, which leads to foam cell formation. To replicate in vivo conditions, we assessed the effect of NaNO3 + DPSC combination therapy on macrophage polarization to M1 and ox‐LDL phagocytosis. It was found that this treatment efficiently reduced M1 macrophage polarization and impeded ox‐LDL ingestion.

The synergistic effect of NaNO_3_ and DPSCs depends critically on the interaction between nitrate‐derived NO and the paracrine activity of DPSCs. Previous research has shown that NO antagonizes redox stress through stimulating HO‐1 gene expression via the Nrf2 pathway [42]. As a master regulator of antioxidant responses, Nrf2 plays a pivotal role in redox signaling and controls the expression of key cytoprotective genes such as HO‐1 [43]. HO‐1, an antioxidant protein highly expressed in endothelial cells, macrophages, and smooth muscle cells [38], has been widely documented to exert anti‐atherosclerotic effects, in part by reducing ROS production and suppressing the development of atherosclerotic lesions [44]. Moreover, activation of the Nrf2 pathway enhances the expression of a spectrum of antioxidant enzymes, including SOD, thioredoxin peroxidase (Tpx), and CAT, thereby providing comprehensive protection against oxidative stress and its downstream sequelae, such as inflammation, apoptosis, and cellular dysfunction [45, 46]. Consistent with these mechanisms, our in vitro experiments demonstrated that the combination of NaNO_3_ and DPSC‐CM significantly suppressed H_2_O_2_‐induced ROS generation and upregulated eNOS expression. We further evaluated the expression of Nrf2, and its downstream antioxidant proteins—HO‐1, MnSOD, and GPX4—in endothelial cells under oxidative stress using Western blot analysis. The results revealed that the combination treatment significantly upregulates the expression of Nrf2, HO‐1, MnSOD, and GPX4, indicating a reinforcement of the cellular antioxidant defense system. These findings suggest that NO derived from NaNO_3_ activates the Nrf2 pathway in DPSC, stimulating the secretion of HO‐1, MnSOD, and GPX4, which collectively enhance cryoprotection against oxidative injury. This positive regulatory loop may amplify the antioxidative and anti‐atherosclerotic efficacy of the combined therapy. Although the potential contribution of nitrolipid formation to HO‐1 induction in the HFD model was not directly examined in this study, it may represent an additional mechanism. Nevertheless, our experimental data demonstrating Nrf2 transcriptional activation and HO‐1 protein upregulation, together with previous evidence from Rudolph et al. [47] and Cole et al. [48], strongly support the conclusion that the co‐administration of NaNO_3_ and DPSC alleviates AS through coordinated modulation of oxidative stress and inflammatory responses, in which the Nrf2/HO‐1 pathway serves as a central mediator. The details of these mechanisms will be addressed in subsequent researches.

## 5. Conclusion

This study demonstrated that the combination of NaNO_3_ and DPSC significantly attenuates vascular oxidative stress in both atherosclerotic mice and cellular models, largely through activation of the Nrf2/HO‐1 pathway. NaNO3 and DPSC acted synergistically to enhance antioxidant defense and mitigate key atherogenic processes. It was the first demonstration that they can alleviate AS by suppressing oxidative stress and diminishing macrophage M1 polarization, offering a novel therapeutic approach for AS.

## Author Contributions

**Zhuang Mao and Han Duan:** investigation, validation, writing – original draft. **Zhichao He and Hu Cao:** investigation, methodology. **Lin Lv and Jingyuan Shao:** formal analysis. **Haichao Yu:** investigation. **Xuesong Zhang:** conceptualization, funding acquisition. **Shunying Hu:** conceptualization, writing – review and editing. **Hua Wang:** conceptualization, supervision, validation, writing – review and editing.

## Funding

This work was supported by the Beijing Municipal Natural Science Foundation (Grant 7232167).

## Ethics Statement

All animal experiments were approved by the Institutional Animal Care and Use Committee of Laboratory Animal Centre (IACUC‐DWZX‐2022‐732).

## Conflicts of Interest

The authors declare no conflicts of interest.

## Data Availability

The data that support the findings of this study are available from the corresponding author upon reasonable request.
